# Individual determinants associated with utilisation of sexual and reproductive health care services for HIV and AIDS prevention by male adolescents

**DOI:** 10.4102/curationis.v41i1.1806

**Published:** 2018-07-31

**Authors:** Omari Shabani, Mary M. Moleki, Gloria G.B. Thupayagale-Tshweneagae

**Affiliations:** 1 Department of Health Studies, University of South Africa, South Africa

## Abstract

**Background:**

Adolescent sexual and reproductive health is one of the essential health care programmes in the world. However, adolescents still face numerous challenges in the area of sexual and reproductive health, which hinder their utilisation of available Sexual and Reproductive HealthCare Services (SRHCS). Male adolescents face further obstacles in accessing and utilising sexual reproductive health services owing to the influence of social constructions of masculinity, which has a bearing on how they view sexual and reproductive health services and their use.

**Objectives:**

The aim of this study was to investigate individual determinants associated with utilisation of SRHCS for HIV and AIDS prevention by male adolescents.

**Method:**

An exploratory, descriptive and contextual qualitative design was used and semi-structured interviews with 20 purposively selected male adolescents aged 18–24 years living in the South African seat of government were conducted. Data were analysed using Tesch’s approach of data analysis.

**Results:**

Knowledge of existing services was described as a significant individual determinant of utilisation of SRHCS. This was linked to the quality of SRHCS and violation of human rights of male adolescents.

**Conclusion:**

The study recommends the development of a strategy that will enhance and promote the utilisation of SRHCS by male adolescents.

## Introduction

Adolescent Sexual and Reproductive HealthCare Services (SRHCS) have been overlooked even in the Sustainable Development Goals promoted by the United Nations (United Nations Fund for Population Activities [UNFPA] 2016). Indeed, it is only recently that international organisations such as World Health Organization (WHO) and United Nations Children’s Fund (UNICEF) began to concentrate on the programmes that promote adolescent SRHCS. These services are aimed at the achievement and promotion of both sexual and reproductive health by public health care providers or professionals to the public or the community. Sexual and Reproductive HealthCare Services constitutes a significant area of the global burden of sexual health challenges (UNFPA 2016).

The term ‘adolescence’ is a construct used to define the transitional phase between childhood and adulthood. The length of this period may range from very short in rural societies (or almost non-existent) where an early marriage or union is expected as soon as a person has joined the workforce to an extended period of 10 years or more in urban societies (Pan American Health Organization 2010). World Health Organization’s well-known definition of adolescence is the period from 10 to 19 years of age, although it is widely acknowledged that adolescence has different meanings and may occur at different chronological ages in different social and economic settings. An adolescent is therefore a young person who is developing into an adult, a young person who is going through adolescence. In this study, adolescents refer to males who are between the ages of 18 and 24 years.

The utilisation of SRHCS refers to the ability to access and make use of, in an appropriate and convenient manner, the SRHCS that are available in a particular area. These services are able to provide a state of physical, mental and emotional well-being, and not merely the absence of disease, in aspects related to sexuality and reproduction. Awoyemi, Obayelu and Opaluwa (2011) assert that health care utilisation is the use of health care services by people. The health care utilisation of a population is related to the availability, quality and cost of services, as well as to the socio-economic structure and personal characteristics of the users. Sexual and Reproductive HealthCare Services are aimed at the achievement and promotion of both sexual and reproductive health by public health care providers or professionals to the public or community, in order to reach a state of physical, mental and emotional well-being in all aspects related to sexuality and reproduction.

Adolescents face numerous challenges that hinder utilisation of available SRHCS (Ramkissoon et al. 2010). Such challenges include fewer services available, lack of information, absence of Youth Friendly Services (YFSs), stigma and discrimination, stereotypes, no services designed for male adolescents, lack of awareness and affordability of the services (Buzi & Smith 2014). Sexual and Reproductive HealthCare Services are aimed at making the services relevant for adolescents, hence the coining of the term ‘Youth Friendly Services’. Youth Friendly Services are ‘health services that do not discriminate or intimidate and that are accessible, acceptable, affordable, and appropriate for adolescents and young people’ (WHO 2012). Regardless of this, services for youth are reported to be underutilised and adolescents continue to face many challenges.

Such challenges have led adolescents to experience many sexual and reproductive health problems, which include potential risk factors, such as sexually transmitted infections (STIs), HIV and AIDS, unwanted pregnancies, unsafe abortions, lack of contraception, sexual abuse (including rape), female genital mutilation and maternal child mortality (Kleep, Alan & Sylvia 2008). In response to these challenges and obstacles, SRHCS for adolescent programmes have emerged as an area of key concern. This is particularly true in developing nations and regions, such as sub-Saharan Africa, where HIV and AIDS account for the second highest number of deaths and one-fourth of these cases represent people under the age of 25 years. Sixty-three per cent of these young people reside in sub-Saharan Africa and in South Africa, where more than 60% of people affected by HIV and AIDS are young people aged between 15 and 45 years (Kleep et al. 2008).

Many countries and scholars in the area of SRHCS have developed a plethora of strategies in a bid to determine why adolescents do not use SRHCS services available to them. However, many factors determine the utilisation of SRHCS by adolescents. Such factors include socio-economic determinants, physical environment and individual determinants.

Generally, utilisation of health care services is influenced by three categories of factors, namely the societal, the health services system and the individual determinants (Anderson & Newman 2005). Individual determinants such as age, past illness and others contribute to utilisation or lack of utilisation of health care services (Anderson & Newman 2005).

Underutilisation of SRHCS by male adolescents compared to female adolescents is even higher (Marcell, Wibbelsman & Seigel 2011). The underutilisation of SRHCS by male adolescents is attributable to many reasons, including the fact that the services are tailored for female adolescents rather than males. Other reasons may include delayed onset of puberty and the traditional masculine beliefs that preclude them from seeking care (Ayehu, Kassaw & Hailu 2016). It is against this background that this study focuses on male adolescents’ utilisation of SRHCS from the vantage point of individual determinants.

## Problem statement

Adolescents and youth have the highest incidence of HIV and AIDS-related conditions (UNAIDS 2016; WHO 2014, 2015). This is despite the existence of SRHCS for adolescents. Studies have also alluded to underutilisation of available SRHCS for adolescents services, especially by male adolescents. Most of the SRHCS services are tailored for female adolescents and male adolescents are passive recipients of the services. Sexual reproductive challenges that occur such as teen pregnancy and high HIV and AIDS infections affect both male and female adolescents, and yet male adolescents are not fully catered for. This is in line with the findings of Ramkissoon et al. (2010) who state that the sexual and reproductive health needs and the rights of men are generally not addressed in the public sector health services where male reproductive health services are largely absent.

## Objectives of the study

The main objective of this study was to investigate the individual determinants associated with utilisation of SRHCS for HIV and AIDS prevention by male adolescents.

## Significance of the study

Understanding the individual determinants that influence utilisation of SRHCS by adolescents will assist policymakers and health personnel to react appropriately to this need. Therefore, male adolescents could propose new interventions that might help improve utilisation of the SRHCS. The high incidence rates of HIV and AIDS among adolescents may also be reduced given the understanding of factors that hinder the use of SRHCS.

## Methods

An explorative, descriptive and contextual qualitative design was utilised to investigate the individual determinants associated with male adolescents’ utilisation of SRHCS for HIV and AIDS prevention. Qualitative approaches allowed the researchers to interact with participants in order to gain deeper understanding of the phenomenon being studied and to view the findings in the context of the respondents’ worldview (Polit & Beck 2012).

### Sample and setting

This study was conducted in the Tshwane Metropolitan Municipality. The choice of this setting was informed by the following factors: (1) a higher prevalence of HIV (11.7% in 2012) in the Tshwane Metropolitan Municipality, (2) male adolescents’ underutilisation of the available SRHCS as discovered by the researcher’s investigation and observation, (3) the lack of SRHCS designed specifically for males, (4) the scarcity of studies on male adolescents and their utilisation of the available SRHCS, (5) male adolescents are not often mentioned as an at-risk group and (6) convenience of the researcher who lives in the same municipality. The researcher holds a view that focusing on male adolescents and educating them appropriately and ensuring they use available facilities would empower them and make them responsible adult males.

A non-probability purposive sampling technique was used to recruit participants (male adolescents) who were aged 18 to 24 years living in the municipality and willing to take part in the study. The researcher involved colleagues, friends and supervisors to recruit the research participants. The recruitment was done by means of telephone calls to set up appointments for briefings as well as the interviews. Because of the difficulty in getting the research participants, the briefing sessions were done not in a group but with each and every participant separately. During the sessions, the participants were informed of the aim, objectives of the study, their legal rights and what would happen to the data (information) they would provide; they were also requested to sign the informed consent form indicating their willingness and acceptance to participate in the study.

### Data collection

Data were collected between January and August 2016. The first author, who at the time was a doctoral candidate, collected data. Data were collected in the assigned room in the clinic, which was normally used for counselling clients who came to the clinic. The room was private and it did not raise any suspicions, as it was a normal clinic room used in the clinic. However, two participants asked to be interviewed in a mall at a date and time agreed by the researcher because they were in a hurry to go somewhere. The two participants met their obligation and were later interviewed over tea. Semi-structured individual interviews lasting between 30 min and 45 min were conducted. Interviews were audiotaped with the participants’ permission. A common question was: tell me what determines utilisation of SRHCS for HIV and AIDS services by male adolescents? Many probes followed this question depending on the response. This was in accordance with the guidelines proposed by Hennink, Hutte and Bailey (2011).

### Trustworthiness

Strategies to enhance trustworthiness (credibility, transferability, dependability and confirmability) were explored. They included the adoption of well-established research methods, the provision of detailed description of the research process, the use of purposive sampling technique, the documentation of real life experiences and real personal stories of participants, the substantiation of the report of the interviews by reviewing similar studies previously conducted and prolonged engagement with male adolescents. The first author visited the clinics once a week for 3 weeks before data collection. Reliability of the data was ensured by involving all authors in the transcription and cross validation of the data. The researcher reflected about the impact of his role, personal background, culture on the study before, during and after the study and has prevented and avoided any biases thereof.

### Data analysis

All the data collected in the field in the form of voice recordings were transcribed verbatim and later analysed using Tesch’s method of data analysis (Creswell 2013). Two researchers, independent of one another, completed the process of data analysis. The third author checked a sample of three transcripts to verify their correctness. Data were listed and grouped into preliminary groupings of descriptive themes agreed upon by the two researchers during a consensus discussion meeting.

### Ethical considerations

Ethical clearance (HSHDC/245/2013) was sought and granted by the ethics committee of the Department of Health Studies of the University of South Africa. Permission to conduct the study in the Tshwane Metropolitan Municipality was also sought and granted by the Tshwane Metropolitan Municipality (Health and Social Development Department Multisectoral AIDS Management Unit). All participants signed the consent form before data collection, indicating their willingness and acceptance to take part in the study. The following ethical considerations pertaining to the study were adhered to: informed consent, voluntary participation, confidentiality and participants’ rights to autonomy, participants’ rights to self-determination, participants’ rights to privacy, participants’ rights to fair treatment, avoidance of harm and responsibility to seek advice.

## Results

The results are presented in line with the study objective. Three key themes emerged: Knowledge of SRHCS for adolescents, poor quality of SRHCS for adolescents and violation of human rights. Themes and sub-themes are presented in Table 1.

**TABLE 1:** Themes and sub-themes.

No.	Themes	Sub-themes
1.	Knowledge of adolescent sexual and reproductive health services (ASRHs)	Lack of knowledge Limited knowledge
2.	Poor quality of ASRHs	-
3.	Violation of human rights	-

### Knowledge of adolescent sexual and reproductive health services

Participants were asked to give their understanding of SRHCS and what these services mean to them. On discussion of issues of knowledge of adolescent sexual and reproductive health services (ASRHs), two sub-themes emerged, namely the limited or lack of knowledge of ASRHs and awareness of available ASRHs.

#### Lack of knowledge

The participants did not know about the existence of specific YFSs and where they could access them. At least 7 out of the 20 participants reported that they just use clinics used by everybody. Some participants (13) talked of their own personal understanding rather than the overall aim of the services. The findings also revealed that the participants lacked interest in services owing to their young age. The participants said the following:

‘I did not know why they are special clinics for the youth and this is why I never went to them and I came to this clinic.’ (P11, male, 19 years, student)

‘I think we can find these services in clinics and hospitals which are available in the city and in the country. I think there may even be a clinic here on campus where they should be offering these services.’ (P6, male, 21 years, student)

‘I don’t have any information about these services. I don’t even know where they are offering them. If I tell you I know where they are I will be lying and also to tell you that they are not being offered, I will be lying. So, I am not sure.’ (P16, male, 19 years, student)

#### Limited knowledge

Participants had limited knowledge as espoused by the extract below:

‘I know they have youth Friendly services for girls only because that is where girls do abortions (laughs). I did not know I can go to them and get condoms.’ (P9, male, 20 years, student)

‘I am clear that if I use condoms I will not contract HIV and for as long as I use a condom I can have as many sexual relations as I want.’ (P4, male, 19 years, student)

‘I heard from my cousin that girls go somewhere for contraceptives and that boys can get condoms in hotels and clinics.’ (P10, male, 20 years, student)

### Poor quality of adolescent reproductive health services

Participants indicated that, in public hospitals and clinics, services were poor with long waiting periods compared to the private sector where services are better. This is what the participants reported:

‘Most of the time I go to pharmacy and buy whatever I need … the service is so fast and quick. There is no queue, so you don’t have to wait for a long time. The service is good and that is why I like going there and that the nurse is so friendly and helpful.’ (P15, male, 21 years, student)

‘I have heard from other youths and friends complaining about the unfriendliness of the health care professionals and more specifically the nurses, the unavailability of doctors, long queues, taking time to be treated and so on. So, for me, I assume that it is because of some of the reasons that the majority of the youth are reluctant to go the hospital. Again as I said before, I have never been at the hospital, these I was told by friends and other people I know.’ (P13, male, 19 years, student)

‘If you go out for HIV test you wait for long queue and for long time waiting to be counselled and it becomes clear to everyone that you may have contracted HIV. It is better to seek help elsewhere.’ (P3, male, 22 years, student)

According to participants, the quality of ASRHs is being undermined by the nurses’ attitudes, lack of respect for patients and unprofessionalism, as well as the lack of privacy. Participants indicated that the negative attitudes of health care professionals such as doctors and nurses towards the youth at different sexual and health care settings, the shortage or unavailability of health care professionals, their lack of commitment and their poor quality (professional training) constitute a very strong barrier to the participants’ utilisation of the services. The respondents indicated that most of the time health care professionals are so hostile to them. Only in few instances, did they receive a friendly treatment espoused by this statement:

‘good morning, how can help you…please sit down. How can I help you? You explain your problem, the nurse will examine you and send you to the Doctor and the pharmacy to get your medications without questioning you or judging you.’ (P20, male, 23 years, student)

### Violation of human rights

Participants indicated that because of the nurses’ attitudes and lack of respect towards them as well as the nurses’ unprofessionalism, they felt stigmatised and discriminated against at SRHCS. Stigmatisation, as used in this study, means the display of nurses and/or health care professionals’ negative and judgmental attitudes towards male adolescents as well as refusal to give me them (male adolescents) attention, care and treatment. This is what they said:

‘I really do not know how to say it but my observation is that there are better services in private clinics than in public clinics and hospitals because of the way the staff treat you. In private clinic, they treat you with respect, dignity and care, in fact like a king. But in public clinics, they see you like a piece of some dirty thing. The healthcare professionals, especially nurses are not professional at all. They do not respect you, they judge you, and they do not care at all.’ (P11, male, 19 years, student)

‘The services are very slow; nurses sometimes even insult you for no reason. One time I went there looking for condoms and when I got there, the person who was on call was a nurse, on entering she asked what I was looking for and when I said that I needed some condoms, instead of giving me condoms, she started lecturing me on how irresponsible I was and she even threatened to tell my parents that I am sexually active and she advised me to do an HIV test because she suspected that I am HIV positive.’ (P8, male, 18 years, student)

‘Does the government think that we men do not need these services or are not supposed to fall sick and cannot fall sick? I think we men are not really considered as human beings. You know even when people talk about social grants; do you know it is not given to men, only to women? Is this not another form of discrimination or marginalisation? So men, don’t have the rights to treatment and other social benefits.’ (P15, male, 21 years, student)

‘The healthcare professionals, especially nurses are not professional at all. They do not respect you, they judge you, and they do not care at all.’ (P12, male, 24 years, student)

Participants considered the lack of services designed specifically for them, the availability of ASRHs designed with females in mind and the lack of consultation by stakeholders in matters regarding their SHR issues as gender discrimination and marginalisation. Thus, making them feel frustrated, discriminated against, marginalised, not considered as human beings with needs and desires, and neglected.

## Discussion

Knowledge of existing services was found to be an important individual determinant of utilisation of SRHCS for adolescents (Kipping et al. 2012). This was linked to the quality of SRHCS for adolescents and violation of human rights (the approach used by health care practitioners in their interaction with male adolescents) of male adolescents. This finding is consistent with the findings of Abajobir and Seme (2014) who found that, to have access and use SRHCS for adolescents, the latter must have enough knowledge regarding how and why the services will be beneficial to them. Advocating an increasing awareness about sexual and reproductive health is also crucial to the success of any adolescent reproductive health effort. Education is a significant social variable affecting reproductive health service utilisation. Kaufman et al. (2016) reported that in the absence of effective education, consultations with peers on facts and advice about sexual health was found to increase responsible sexual health decision-making. And, male adolescents were reported to generally lack awareness and lag behind female adolescents in the knowledge about reproductive health and risks, including STIs and HIV (Kaufman et al. 2016). According to Haberland and Rogow (2015), the International Conference on Population and Development and related resolutions has repeatedly called on governments to provide adolescents and young people with comprehensive sexuality education (CSE).

Poor quality of services was another identified factor that led to underutilisation of SRHCS for adolescents by male adolescents. Poor quality of services was aligned to long waiting periods and attitudes of health care professionals. This finding is not unique to SRHCS for adolescents but a general finding among studies that have looked into poor quality of services (Coleman et al. 2016; Kerr et al. 2015). However, the uniqueness of this finding is that SRHCS for adolescents were meant to be user friendly (WHO 2012). Buttell, Hendler and Daley (2007) define quality of health care as the degree to which health services for individuals and populations increase the likelihood of desired health outcomes and are consistent with current professional knowledge. They also refer to quality as care that meets the expectations of patients and other customers of health care services. According to WHO (2006), quality of life is evaluated by the effectiveness, efficiency, accessibility, acceptability/client-centred, equitability and safe provision of health care services.

In support of the above, Tappis et al. (2015) report that poor quality service provision has been a major barrier to scaling up the use of contraception. Giving information to clients about the benefits and potential side effects of modern methods of contraception is not sufficient. A central goal of health care quality improvement is to maintain what is good about the existing health care system, while focusing on the areas that need improvement (Agency for Healthcare Research and Quality [AHRQ] 2002).

Violation of human rights is one of the themes that emerged from the study. London and Baldwin-Ragaven (2006) define human rights as ‘universally applicable social or material entitlements, essential to fulfil fundamental needs that individuals can claim from society on the basis of their humanity’. The participants in this study experienced lack of respect and breach of confidentiality from the health care workers serving them. Lack of respect is reported in literature as one of the deterrents in accessing services. Stemple (2008) reports that lack of respect for confidentiality of participants adversely affects their use of health care services. Haberland and Rogow (2015) further assert that CSE is a right.

Nair et al. (2015) accentuate that health systems globally have to be responsive to the unique demands of young people and focus on improving quality alongside coverage of youth friendly health care services such that these services are acceptable, effective, efficient, equitable and safe for adolescents. Furthermore, Nair et al. maintain that the evidence from both high- and low-income countries shows that adolescents and young adults face many barriers, which prevent their use of health services. Pockets of excellent practice exist but, overall, services need significant improvement.

The WHO’s (2015) report, ‘Health for the world’s adolescents: a second chance in the second decade’, suggests that to make progress towards universal health coverage, ministries of health and the health sector more generally will need to transform how health systems respond to the health needs of adolescents. It recommends developing and implementing national quality standards and monitoring systems as one of the actions necessary to make this transformation.

## Limitations of the study

The small size of the sample limits generalisation and external validity of the findings as well as the scope of the research. The transferability of the findings is limited owing to the data only being gathered in one area (the study was limited to only the clinics in the Tshwane Metropolitan Municipality). The other limitation was that only participants who spoke English were recruited in the study. The researcher, however, believes that people normally express themselves better in their home language(s). This could then have limited participants’ expression of why they do not utilise the services and even their knowledge of them.

## Recommendations of the study

The study recommends the development of a strategy that will enhance and promote the utilisation of sexual reproductive services by male adolescents for the prevention of HIV and AIDS through the provision of appropriate, accurate and relevant information and knowledge of ASRHs, the improvement of the quality of ASRHs and addressing and dealing effectively and efficiently the issue of violation of human rights of male adolescents.

## Implication for practice

Male adolescents have been excluded in most services with the concern centred mostly on the female adolescents. Male adolescents have a role to play in the reduction of sexual risk behaviours which includes HIV and AIDS prevention and prevention of teen pregnancy. Education and services should be tailored around male adolescents.

Health care service providers should ensure that this strategy is implemented effectively and efficiently by creating a conducive and friendly environment that strives to improve the therapeutic relationships with male adolescents (patients) by adhering to the health care professional code of ethics in order for the latter to make use of the services more frequently and draw benefits.

## Conclusion

The findings of the study revealed a knowledge gap in the utilisation of the available SRHCS by male adolescents. Furthermore, poor quality of services also contributed to poor utilisation of services. Based on the major findings, the study recommends the development of a strategy to enhance and promote the utilisation of SRHCS by male adolescents for the prevention of HIV and AIDS. The strategy to be developed should address the following aspects: limited or lack of knowledge of SRHCS for adolescents, lack of awareness of availability of SRHCS for adolescents, service delivery at the facility (sites), challenges with service providers, stigma and discrimination, violation of code of ethics and the issue of gender discrimination and marginalisation.
